# Genetics and Genomics of Pediatric Neurological Disorders: An Overview of Current Insights and Future Directions

**DOI:** 10.3390/genes17030275

**Published:** 2026-02-27

**Authors:** Antonio Trabacca, Marta De Rinaldis, Maria Carmela Oliva, Ilaria Notaristefano, Ivana Gallo, Camilla Ferrante, Isabella Fanizza

**Affiliations:** 1 Scientific Institute, IRCCS “E. Medea”, Scientific Direction, Via Don L. Monza 20, 23842 Bosisio Parini, LC, Italy; 2 Associazione “La Nostra Famiglia”, IRCCS “E. Medea”, Scientific Hospital for Neurorehabilitation, Unit for Severe Disabilities in Developmental Age and Young Adults (Developmental Neurology and Neurorehabilitation), Piazza “Antonio Di Summa”, 72100 Brindisi, BR, Italy; marta.derinaldis@lanostrafamiglia.it (M.D.R.); mariacarmela.oliva@lanostrafamiglia.it (M.C.O.); ilaria.notaristefano@lanostrafamiglia.it (I.N.); ivana.gallo@lanostrafamiglia.it (I.G.); camilla.ferrante@lanostrafamiglia.it (C.F.); isabella.fanizza@lanostrafamiglia.it (I.F.)

**Keywords:** pediatric neurological disorder, children, inheritance, neuromuscular disorder, epilepsy, neurodegenerative disorder, neurodevelopmental disorder, movement disorder, genetic analysis, whole-exome sequencing, whole-genome sequencing, next generation sequencing

## Abstract

Pediatric neurological disorders comprise a highly heterogeneous group of conditions that together represent a substantial global public health burden. Many have a strong genetic basis and are associated with significant morbidity, premature mortality, and long-term disability, with far-reaching consequences for affected children, their families, and healthcare systems worldwide. Clinical heterogeneity is a hallmark of these disorders, as pathogenic variants in the same gene can give rise to diverse phenotypes with variable severity, age at onset, and disease course. In children, ongoing brain development and somatic growth further complicate diagnosis, often leading to nonspecific or atypical presentations that differ from classical adult neurological phenotypes. Advances in genetics and genomics have fundamentally transformed the understanding, diagnosis, and classification of pediatric neurological diseases. The widespread use of high-throughput sequencing, genome-wide association studies, and integrative bioinformatics approaches has enabled the rapid and precise identification of disease-associated genes, even in sporadic and complex conditions, facilitating earlier and more accurate diagnoses and highlighting the role of genetic background and gene–environment interactions in disease pathogenesis. Here we provide an overview of the genetic and genomic landscape of key pediatric neurological disorders with well-characterized molecular etiologies, including neuromuscular disorders, epilepsies, neurodevelopmental disorders, neurodegenerative diseases, and movement disorders. Current knowledge is synthesized with emphasis on clinical presentation, genetic architecture, and genotype–phenotype correlations. Gene-specific management strategies and emerging precision therapies are discussed for selected conditions, underscoring the central role of genetic diagnosis in guiding clinical decision-making and improving outcomes in affected children.

## 1. Introduction

Pediatric neurological disorders represent a highly heterogeneous group of conditions, many of which have a strong genetic basis. They pose a complex and multifaceted challenge that requires specialized clinical expertise and multidisciplinary care and constitute a substantial global public health burden. These disorders account for a significant proportion of premature mortality and are a leading cause of long-term disability in children, with profound and lasting consequences for affected individuals, their families, and healthcare systems worldwide [1,2,3].

Pediatric neurological disorders are also characterized by marked clinical heterogeneity, as pathogenic variants in the same gene can give rise to distinct syndromes across different individuals, with wide variability in phenotype, symptom severity, age at onset, and disease progression. In contrast to adults, the developing nervous system and ongoing somatic growth in children can mask classical neurological signs, leading to clinical presentations that are often nonspecific, such as hypotonia [4,5].

Advances in genomics have profoundly reshaped our understanding of neurological disease, transforming diagnostic pathways and, in selected cases, therapeutic strategies. Molecular genetics has fundamentally altered, and continues to redefine, the approach to neurological disorders, particularly those affecting children. Ongoing progress in genomic science is yielding deeper insight into human health and disease, with especially significant implications for the diagnosis, classification, and management of childhood-onset neurological conditions. As the extraordinary complexity of the developing brain continues to challenge traditional diagnostic and therapeutic paradigms, advances in genetics provide a powerful framework for uncovering the biological mechanisms underlying a wide spectrum of neurological disorders, ranging from rare neurodevelopmental syndromes to common, multifactorial conditions [6,7].

It is now well-established that even sporadic and complex neurological disorders are influenced, at least in part, by an individual’s genetic background and by dynamic interactions between genes, their products, and environmental factors.

The emergence of advanced genomic technologies has been central to this paradigm shift. High-throughput sequencing platforms, genome-wide association studies, and integrative bioinformatics approaches have revolutionized the identification of disease-associated genes, enabling unprecedented speed and precision [8,9,10].

Collectively, these advances are reshaping pediatric neurology by enabling earlier and more accurate diagnoses, refining disease classification, and paving the way toward targeted and personalized interventions. With the advent of gene-based and precision therapies, the identification of the genetic underpinnings of disease has become increasingly essential for translating genomic discoveries into effective clinical care for children with neurological disorders [11,12,13].

This article provides an overview of the genetics and genomics of key pediatric neurological conditions with well-characterized genetic underpinnings, including neuromuscular disorders, epilepsies, neurodegenerative disorders, neurodevelopmental disorders, and movement disorders. Current knowledge is synthesized with a focus on clinical presentation and genetic architecture, along with mention of gene-specific management strategies for causative variants. For selected conditions, emerging and innovative therapies capable of modifying disease prognosis, clinical course, and natural history are highlighted, underscoring the critical importance of achieving an accurate genetic diagnosis and initiating timely, targeted treatment.

## 2. Methods for Literature Search

This study was designed as a non-systematic, narrative, and pragmatic clinical review of the literature on the genetics and genomics of pediatric neurological disorders. The literature search was conducted using three major scientific databases, NCBI/PubMed, ScienceDirect, and Scopus. Articles were selected based on clinical relevance, scientific quality, and applicability to diagnosis and clinical management. In addition, the selection of specific disorder categories (epilepsies, neuromuscular dis-orders, neurodevelopmental disorders, and movement disorders), as well as representative genes and therapeutic approaches, was guided by complementary criteria, including their prevalence and clinical burden in the pediatric population, the strength of evidence supporting a genetic etiology, the impact of recent advances in genomic technologies on diagnostic pathways, and the availability or emergence of gene-specific or mechanism-based treatments. Particular emphasis was placed on conditions that exemplify broader principles of pediatric neurogenetics, such as genotype–phenotype correlations, diagnostic complexity, and translational implications for precision medicine. Given the differences in genetic characterization, clinical translation, and therapeutic availability across disorders, some sections (e.g., epilepsy and neuromuscular disorders) are more detailed, while others provide a concise overview of emerging concepts and future directions. This approach was chosen to provide a clinically oriented synthesis of a rapidly evolving and multidisciplinary field. A systematic review methodology was intentionally not adopted, as the marked heterogeneity of pediatric neurological disorders, their underlying genetic architectures, and the rapid pace of advances in genomic technologies and therapies render a quantitative and exhaustive synthesis less suitable compared with a narrative, practice-focused overview [14].

## 3. Challenges in Diagnosis and Clinical Management

Over the past decades, there has been a transformative shift in the understanding and management of neurological disorders affecting infants and children, largely driven by major advances in genetics and genomic research. Neurological symptoms are common throughout infancy, childhood, and adolescence and are a frequent reason for clinical evaluation in pediatric practice. Early manifestations, such as hypotonia, developmental delay, seizures, movement disorders, or episodic neurological symptoms, are often nonspecific and may change or evolve over time [15]. This clinical variability makes early recognition of an underlying genetic neurological condition particularly challenging. As a result, phenotypic ambiguity often leads to delayed diagnosis, with children undergoing a prolonged *diagnostic odyssey* involving multiple specialist evaluations before a unifying etiology is ultimately identified [5].

Diagnosis is further complicated by the marked genetic and phenotypic heterogeneity that characterizes pediatric neurological disorders. Pathogenic variants in the same gene may lead to distinct clinical syndromes, resulting in wide variability in phenotype, disease severity, age at onset, and disease progression [16]. In addition, epigenetic mechanisms, including DNA methylation, histone modifications, and noncoding RNAs, as well as genetic modifiers, play a critical role in shaping neurological phenotypes by modulating gene expression and cellular function. Clinicians must also contend with variants of uncertain significance (VUS), incomplete penetrance, and variable expressivity, which pose additional challenges for genetic counseling, therapeutic decisions, and longitudinal patient follow-up [17,18]. A clear example of genotype–phenotype variability is provided by *SCN2A*, in which gain- or loss-of-function variants affecting the voltage-gated sodium channel Nav1.2 are associated with a broad phenotypic spectrum that includes epilepsy, intellectual disability, autism spectrum disorder, episodic ataxia, and schizophrenia, with variable combinations and, in rare cases, overlapping features within the same individual [19]. This example illustrates that many genes implicated in pediatric neurological disorders can lead to overlapping phenotypes across different disease categories, a feature that is clinically relevant for differential diagnosis, guiding genetic testing strategies, and re-fining prognosis.

This phenotypic overlap is further amplified in consanguineous populations, where autosomal recessive (AR) disorders, though less common in outbred populations, represent the predominant genetic architecture, with 82% of causative variants being homozygous and only 13.3% being de novo mutations. In populations with high consanguinity rates such as Turkey (24%), Bangladesh (16%), and regions of North Africa and the Middle East, exome sequencing achieves diagnostic yields of 72–86%, far superior to targeted gene panels that would miss 52% of diagnoses due to extreme genetic heterogeneity and overlapping phenotypes characteristic of AR neurogenetic diseases. The risk of genetic abnormality or death in early childhood is approximately 5% in children of consanguineous couples versus 2–2.5% in non-consanguineous couples, underscoring the importance of population-specific diagnostic strategies that account for distinct modes of inheritance and their associated phenotypic patterns [20,21,22,23].

A major challenge in the interpretation of genomic data lies in the extensive background of genetic variation in the general population, including numerous benign polymorphisms that can be difficult to distinguish from pathogenic variants. Consequently, the clinical relevance of many detected variants remains uncertain at the time of initial analysis. Furthermore, the proportion of de novo versus inherited mutations varies across neurogenetic disorders and has direct implications for genetic counseling and recurrence risk assessment. The proportion of familial versus sporadic cases varies significantly across genes, reflecting differences in inheritance patterns and phenotypic severity. For Duchenne muscular dystrophy (DMD), approximately 30% of cases arise de novo, while 70% are inherited, consistent with Haldane’s rule for X-linked disorders; germline mosaicism occurs in 7–14% of cases, with implications for genetic counseling. In contrast, *SCN1A*-related Dravet syndrome shows a predominance of de novo mutations (~90%), with only 10% of cases inherited from parents with somatic mosaicism or milder phenotypes such as generalized epilepsy with febrile seizures plus (GEFS+). Similarly, *MECP2* mutations causing Rett syndrome are de novo in over 95–99% of cases, almost exclusively of paternal origin, with familial cases being exceptionally rare due to male lethality. For autosomal recessive conditions such as *SMN1*-related spinal muscular atrophy (SMA), the pattern is reversed: approximately 98% of cases are inherited from carrier parents, with de novo mutations accounting for only 2%. *TSC1*/*TSC2* mutations in tuberous sclerosis complex are sporadic in approximately two-thirds of cases, with *TSC2* mutations more frequent among sporadic presentations. More broadly, de novo mutations are recognized as a major cause of neurodevelopmental disorders, accounting for approximately 80% of genetically resolved cases and contributing to an estimated 15% of epilepsy, autism spectrum disorder (ASD), and neuromotor impairments [24,25,26,27]. Addressing this uncertainty requires a rigorous and structured clinical approach, beginning with a detailed medical history, followed by a comprehensive physical and neurological examination [28]. Phenotypic evaluation should integrate information across multiple domains, taking into account developmental trajectories and brain–behavior relationships, including the interplay between cognitive functioning, medical comorbidities, and psychiatric features [29]. Careful consideration of patterns of genetic inheritance, pedigree analysis, and modifying factors that influence disease expression is also essential [30]. In this context, longitudinal clinical evaluation becomes particularly important, as repeated assessments and follow-up visits allow clinicians to document the evolution of clinical features and progressively refine genotype–phenotype correlations [29,31].

Effective interpretation of genomic findings therefore depends on the integration of variant-based analytical approaches with detailed, systematic, and evolving phenotypic assessment. This integrative process is optimally achieved within a multidisciplinary team setting, in which pediatric neurologists, clinical geneticists, bioinformaticians, and other specialists collaboratively interpret genomic data in light of comprehensive and longitudinal clinical information. Such multidisciplinary team setting discussions are critical for establishing coherent and clinically meaningful genotype–phenotype associations, ultimately informing diagnosis, prognosis, and patient management [32,33,34,35].

## 4. Genomic and Molecular Advances in Pediatric Neurology

Since the discovery of the structure of DNA in 1953 by James Watson and Francis Crick, which laid the foundation for understanding heredity, genetic mutation, and replication, the concept of genetic disease has evolved profoundly [36]. Whereas disorders were once broadly categorized as either “genetic” or “non-genetic”, the completion of the Human Genome Project in 2003 marked the beginning of a new era in molecular medicine [37]. It is now well-established that even sporadic and complex neurological disorders are influenced, at least in part, by an individual’s genetic background, together with dynamic interactions between genes, their products, epigenetic regulation, and environmental factors [38].

Earlier targeted molecular approaches developed in the 1970s and 1980s, most notably polymerase chain reaction (PCR) and DNA sequencing methods pioneered by Frederick Sanger, enabled the identification of pathogenic variants in monogenic pediatric conditions, including epileptic encephalopathies, neuromuscular disorders, and neurodevelopmental syndromes [39,40]. These methodologies established the first robust gene-disease associations and laid the groundwork for a molecular classification of disorders that had previously been defined exclusively by clinical criteria.

A major paradigm shift occurred with the introduction of next-generation sequencing (NGS) technologies in the mid-2000s, which enabled massively parallel sequencing of millions to billions of DNA fragments within a single experiment [41]. This technological advance dramatically reduced costs and turnaround times, making comprehensive genomic analysis feasible in routine clinical practice. Targeted gene panels, whole-exome sequencing (WES), and whole-genome sequencing (WGS) are now central tools in pediatric neurological disorders. Together with RNA sequencing (RNA-Seq), these approaches allow the detection of rare inherited variants, de novo mutations, copy number and structural variants, noncoding and regulatory alterations, and complex genetic architectures, including oligogenic and polygenic contributions to disease [42,43]. The widespread adoption of these NGS-based techniques has substantially increased diagnostic yield, refined genotype–phenotype correlations, and significantly reduced the diagnostic odyssey for affected children and their families.

More recently, emerging multi-omic approaches, including single-cell and spatial transcriptomics, have enabled detailed characterization of cellular heterogeneity and cell-type-specific vulnerability within the developing brain, providing critical insight into the timing and context of genetic perturbations. Integration of genomic data with proteomic, metabolomic, and epigenomic analyses further enhances the understanding of regulatory networks, signaling pathways, and metabolic processes underlying neurodevelopmental and neurodegenerative disorders in childhood [44].

Collectively, these advances provide a multidimensional framework for elucidating disease mechanisms and have accelerated the translation of precision medicine into pediatric neurology. Increasingly, genomic insights inform prognosis, guide surveillance strategies, and enable access to gene-based and molecularly targeted therapies, underscoring the central role of early and accurate genetic diagnosis in improving clinical outcomes for children with neurological disorders.

## 5. Pediatric Neurological Disorders

### 5.1. Epilepsies

Epilepsy is one of the most common neurological disorders in childhood, with an estimated prevalence of approximately 0.5–1% in the pediatric population [45,46]. A substantial genetic contribution underlies epilepsy susceptibility, with heritable factors estimated to account for 70–80% of overall disease risk. While approximately 30% of this risk in generalized epilepsies can be attributed to common genetic variants, a significant proportion arises from rare, often highly penetrant variants. Consequently, the genetic and genomic architecture of pediatric epilepsy is highly heterogeneous, encompassing both monogenic and polygenic mechanisms that influence disease susceptibility, age at onset, clinical presentation, severity, and response to treatment [47]. These genetic determinants interact with environmental factors and neurodevelopmental processes, shaping disease onset and progression within the dynamic context of the developing brain [48,49,50]. Genetic epilepsies may follow Mendelian inheritance, with pathogenic variants transmitted in an autosomal dominant, autosomal recessive, or X-linked manner, or arise through non-Mendelian mechanisms such as de novo variants, somatic mosaicism, or mitochondrial genetic alterations. Monogenic epilepsies account for approximately 10–30% of all epilepsy cases and are particularly prevalent in pediatric populations, where they often present as severe, early-onset disorders [51,52,53]. These conditions result from pathogenic variants in single genes involved in neuronal excitability, synaptic transmission, and neurodevelopmental pathways. To date, more than 1000 genes have been implicated as monogenic causes of epilepsy, reflecting the complexity of epilepsy pathogenesis across molecular, cellular, and network levels [54,55]. Among pediatric epilepsies, developmental and epileptic encephalopathies (DEEs) represent the most severe group, with seizure onset typically occurring in infancy or early childhood. DEEs are defined by the coexistence of a primary developmental encephalopathy, which disrupts cognitive and developmental trajectories, and an epileptic encephalopathy, in which epileptic activity itself contributes to developmental slowing or regression. The etiological landscape of DEEs is highly heterogeneous and varies across geographical regions, although most available data derive from high-resource settings [56,57]. Current genetic studies identify a causative diagnosis in up to approximately 50% of affected individuals, most commonly involving de novo, autosomal recessive, or X-linked pathogenic variants. DEEs may arise from structural brain abnormalities or from pathogenic variants in one or more genes, with over 900 genes reported to be associated with these disorders. These genes encode proteins involved in diverse biological pathways, including ion channels and transporters (e.g., *SCN1A*, *SCN8A*, *KCNA1/2*), synaptic proteins (e.g., *SYNGAP1*, *CNTNAP2*), cell signaling pathways (notably the mTOR pathway), and epigenetic regulation (e.g., *CDKL5*, *CHD2*) [48,57,58,59,60]. A prototypical DEE is Dravet syndrome, first described in 1978, which is caused in more than 90% of cases by heterozygous loss-of-function pathogenic variants in *SCN1A*, encoding the voltage-gated sodium channel NaV1.1 [61,62]. In most individuals, these variants arise de novo. Pathogenic variants in additional genes, including *SCN2A*, *SCN1B*, *GABRA1*, *GABRB3*, *ALG13*, *GABRG2*, *CDKL5*, *PCDH19*, *STXBP1*, *HCN1*, and *CHD2*, have been associated with Dravet-like or overlapping phenotypes, further expanding the genetic spectrum of DEEs [61,63,64]. The most frequent X-linked DEEs include *CDKL5* deficiency disorder, *MECP2*-related Rett syndrome, and *PCDH19*-related epilepsy; notably, some individuals with Rett syndrome or *PCDH19*-related epilepsy exhibit a milder seizure burden and may be more appropriately classified as having developmental epilepsy rather than DEE [65]. Beyond DEEs, monogenic epilepsies encompass a wide range of additional phenotypes [66,67,68]. X-linked neuronal migration disorders include periventricular nodular heterotopia associated with *FLNA* variants and double cortex or subcortical band heterotopia caused by pathogenic variants in DCX. Pathogenic variants in the potassium channel genes *KCNQ2* and *KCNQ3*, encoding the voltage-gated potassium channels KV7.2 and KV7.3, are associated with a broad clinical spectrum ranging from transient benign familial neonatal seizures to severe, long-lasting DEEs [69,70,71,72]. Focal epilepsy syndromes may also result from pathogenic variants in multiple genes, including *DEPDC5*, *KCNT1*, and *GRIN2A* [73,74,75,76]. In particular, *DEPDC5* variants represent a major cause of both sporadic and familial focal epilepsies, including autosomal dominant sleep-related hypermotor epilepsy, autosomal dominant epilepsy with auditory features, benign epilepsy with centrotemporal spikes, familial focal epilepsy with variable foci, and infantile spasms. Loss-of-function variants in *DEPDC5* disrupt the *GATOR1* complex, leading to hyperactivation of the mTORC1 pathway and contributing to focal epileptogenesis [77,78]. Overall, *DEPDC5*-related epilepsies display marked clinical and genetic heterogeneity. In contrast to monogenic epilepsies, polygenic epilepsies arise from the cumulative effect of hundreds to thousands of common genetic variants, each conferring a small increase in disease risk [79]. These forms do not follow Mendelian inheritance patterns and are characterized by lower penetrance at the individual variant level but higher prevalence in the general population. Large genome-wide association studies have identified multiple risk loci, including variants in *SCN1A*, *GABRA2*, and *CACNA1H*, highlighting their contribution to common epilepsy phenotypes such as genetic generalized epilepsies and non-lesional focal epilepsies [80]. The aggregate impact of these variants, referred to as the polygenic burden, can be quantified using polygenic risk scores; however, despite their promise for risk stratification and mechanistic insight, polygenic risk scores are not yet ready for routine clinical use in epilepsy [81]. Genetic diagnoses in pediatric epilepsy have a substantial impact on clinical management, leading to changes in care in approximately 12–80% of cases, depending on the cohort studied [82]. Genetic information increasingly informs therapeutic decisions by enabling the selection or avoidance of specific antiseizure medications based on underlying molecular mechanisms. Examples include the use of fenfluramine and stiripentol and avoidance of sodium channel blockers in *SCN1A*-related Dravet syndrome; treatment with everolimus in tuberous sclerosis complex; and implementation of the ketogenic diet in *SLC2A1* (*GLUT1* deficiency syndrome) [83,84]. Genetic diagnoses also support drug repurposing strategies, such as memantine for gain-of-function variants in *GRIN2A* and quinidine for gain-of-function variants in *KCNT1*, and facilitate access to precision medicine clinical trials [59,85]. An antisense oligonucleotide trial is currently enrolling individuals with Dravet syndrome, with additional nucleotide-based therapies in development [86]. Finally, genetic testing provides important prognostic information, particularly in neurodegenerative epilepsies, and may predict seizure evolution or the later emergence of additional neurological features, as observed in *SCN1A*, and *STXBP1* related disorders [87,88].

### 5.2. Neuromuscular Disorders

Inherited neuromuscular disorders (NMDs) in childhood constitute a broad and heterogeneous group of genetic conditions affecting the anterior horn cell motor neuron, peripheral nerve, neuromuscular junction, or skeletal muscle. These disorders affect 1 in 2000 children worldwide [15,89]. The vast majority of pediatric neuromuscular disorders have a genetic basis, most often caused by inherited autosomal dominant, autosomal recessive, or X-linked variants, as well as de novo pathogenic variants in single genes. These conditions are typically progressive, leading to motor impairment, reduced quality of life, and, in many cases, shortened life expectancy. Although many of these disorders were clinically recognized more than a century ago, their molecular underpinnings have been largely elucidated over the past three decades, profoundly transforming diagnostic approaches, disease classification, and therapeutic strategies [90]. The identification of disease-causing genes has markedly improved diagnostic accuracy and deepened the understanding of pathogenic mechanisms while also enabling the development of targeted and disease-modifying therapies. Advances in molecular genetics over the last decade have been particularly impactful in pediatric populations, supporting the widespread adoption of blood-based genetic testing as a first-line diagnostic tool. For many children, molecular testing has reduced or eliminated the need for invasive diagnostic procedures such as muscle or nerve biopsy and has helped avoid unnecessary exposure to immunosuppressive treatments and their associated complications. These advances have also promoted a shift toward phenotype-driven, targeted genetic testing strategies, sometimes applied sequentially, rather than indiscriminate use of broad gene panels. Early and accurate molecular diagnosis is especially critical in childhood, when timely intervention can meaningfully alter disease trajectory and improve long-term outcomes [91].

#### 5.2.1. Anterior Horn Cell Disorders

Inherited anterior horn cell disorders in childhood are most commonly caused by mutations in the *SMN1* gene on chromosome 5q13, resulting in spinal muscular atrophy (SMA). SMA is a rare autosomal recessive disorder characterized by progressive muscle weakness and atrophy due to the degeneration of spinal α-motor neurons and, in more severe forms, lower bulbar motor neurons. With an estimated prevalence of approximately 2 per 100,000 individuals, SMA represents the most frequent genetic cause of infantile motor neuron disease [92,93]. Clinically, SMA encompasses a phenotypic continuum traditionally classified into five subtypes (types 0–4), distinguished by age at onset, disease severity, and maximum motor function achieved. Types 1, 2, and 3 account for the majority of pediatric cases, ranging from severe infantile-onset disease to milder childhood-onset forms [93,94]. At the molecular level, SMA is caused by homozygous deletions, mutations, or rearrangements of *SMN1*, leading to deficiency of the ubiquitously expressed survival motor neuron (SMN) protein. Humans uniquely harbor a paralogous gene, *SMN2*, which differs from *SMN1* by a critical nucleotide change in exon 7 that results in exon skipping in most transcripts and the production of a truncated, unstable protein. Consequently, *SMN2* produces only limited amounts of full-length functional SMN protein and partially compensates for *SMN1* loss. Importantly, *SMN2* copy number is a key modifier of disease severity, with higher copy numbers associated with milder phenotypes, accounting for the broad clinical variability observed in SMA [95].

Notably, therapeutic advances have transformed the clinical landscape of SMA. Three gene-based therapies, nusinersen and risdiplam (RNA-based therapy) and onasemnogene abeparvovec (DNA-based therapy), have recently been authorized and have dramatically improved survival and motor outcomes, particularly when initiated early, underscoring the critical importance of timely genetic diagnosis in affected infants and children [96,97].

#### 5.2.2. Peripheral Nerve Disorders-Neuropathies

Hereditary motor and sensory neuropathies (HMSNs), collectively referred to as Charcot-Marie-Tooth (CMT) disease, represent the most common inherited disorders of the peripheral nervous system and account for up to approximately 40% of chronic neuropathies presenting in childhood [98]. CMT is the most prevalent inherited neuromuscular disease, with reported population prevalence ranging from 9.4 to 82 per 100,000 depending on geographic region and ethnicity [99,100]. Clinically, CMT is characterized by slowly progressive distal muscle weakness and atrophy, typically associated with areflexia, sensory loss, and characteristic foot deformities such as pes cavus and hammertoes. Sensory symptoms are often mild, which may delay recognition and diagnosis into adolescence or adulthood despite childhood onset. CMT disorders exhibit marked genetic and phenotypic heterogeneity, with autosomal dominant, autosomal recessive, and X-linked inheritance patterns, and are broadly classified into demyelinating and axonal forms [101,102]. Variants in *PMP22*, *GJB1*, *MPZ*, and *MFN2* account for the majority of genetically defined cases. Alterations involving *PMP22* are the most frequent molecular cause: duplications of *PMP22* are typically associated with CMT1A, whereas deletions resulting in *PMP22* haploinsufficiency cause hereditary neuropathy with liability to pressure palsies (HNPP) [103,104]. Axonal forms of CMT (CMT2) typically have later onset, often in the second or third decade, and display substantial genetic heterogeneity [105]. X-linked CMT, most commonly caused by *GJB1* mutations, often presents with gait instability, frequent falls, distal weakness, muscle atrophy, and areflexia, with females generally exhibiting milder phenotypes and later onset [106]. Notably, even among cohorts of individuals with previously unexplained axonal neuropathies in mid-to-late adulthood, disease-causing variants are identified in a significant proportion of cases through genomic testing, underscoring the diagnostic value of molecular analysis across the lifespan.

As the number of genes associated with CMT continues to expand, the diagnostic yield of genetic testing has steadily increased [107]. Targeted gene panel testing identifies pathogenic or likely pathogenic variants in approximately 15–20% of cases in selected cohorts, while more comprehensive strategies integrating chromosomal microarray, gene panel sequencing, and whole-exome sequencing have achieved diagnostic yields exceeding 30% in large, population-based studies [108]. These findings highlight the importance of stepwise and comprehensive genetic testing approaches, including copy number analysis, for accurate diagnosis, genetic counseling. In parallel, a range of therapeutic strategies is currently under investigation, aiming to regulate PMP22 expression and enhance myelination, supporting future gene-informed management of CMT and related neuropathies [109].

#### 5.2.3. Disorders of the Neuromuscular Junction

Congenital myasthenic syndromes (CMSs) comprise more than 30 genetically distinct disorders caused by impaired neuromuscular transmission and characterized by fatigable muscle weakness, typically presented in infancy or early childhood, although later onset forms also occur. Clinical features include fluctuating weakness, abnormal fatigability, muscle atrophy, and, in some cases, mild facial or skeletal anomalies. CMSs result from pathogenic variants in genes encoding key components of the neuromuscular junction, including subunits of the nicotinic acetylcholine receptor (AChR), *rapsyn*, and elements of the *agrin-LRP4-MuSK-Dok-7* signaling pathway responsible for AChR clustering and synapse maintenance [110,111]. Additional genes involved in synaptic transmission and muscle excitability include *SCN4A*, encoding the voltage-gated sodium channel Nav1.4, as well as *COLQ* and *CHAT*, which are essential for neuromuscular junction function. In addition, mutations in *HSPG2*, encoding perlecan, cause Schwartz–Jampel syndrome. Advances in next-generation sequencing have expanded the genetic spectrum of CMSs and enabled precise molecular diagnosis, which is essential for management. Treatment response is genotype dependent, and precision therapies, including acetylcholinesterase inhibitors, sympathomimetic agents, and channel modulators such as fluoxetine or quinidine, may be beneficial or harmful depending on the underlying molecular defect. These disorders highlight the critical role of genetic stratification in guiding personalized therapy for pediatric neuromuscular disease [112].

#### 5.2.4. Primary Muscle Diseases

Primary muscle diseases comprise a genetically and clinically heterogeneous group of inherited neuromuscular disorders, including dystrophinopathies, limb-girdle muscular dystrophies, myotonic dystrophies, congenital myopathies, and metabolic myopathies. Advances in molecular genetics have revealed that these conditions, despite their diverse clinical presentations, are unified by pathogenic variants affecting proteins involved in muscle fiber integrity, excitation–contraction coupling, intracellular signaling, RNA processing, and metabolic homeostasis, thereby reshaping diagnostic pathways and therapeutic strategies [113,114,115]. Dystrophinopathies represent a paradigmatic example of genotype–phenotype correlation in pediatric neuromuscular disease. Duchenne and Becker muscular dystrophies are caused by pathogenic variants in the *DMD* gene on chromosome Xp21, which encodes dystrophin, a key component of the dystrophin–glycoprotein complex required for sarcolemmal stability. The exceptional size of the *DMD* gene, spanning 79 exons, results in marked mutational heterogeneity. Approximately 68–70% of pathogenic variants are large exon deletions, 8–11% are duplications, and 18–20% consist of small mutations, including insertions, deletions, and single-nucleotide substitutions affecting exonic and intronic regions [116]. Frame-disrupting variants are typically associated with Duchenne muscular dystrophy, the most common childhood muscular dystrophy, characterized by early-onset progressive weakness and cardiopulmonary involvement, whereas in-frame variants often result in the milder Becker phenotype [117,118]. These molecular distinctions have direct clinical relevance, as genetic characterization informs prognosis and eligibility for mutation-specific therapies [119]. Although dystrophinopathies primarily affect males, symptomatic females may occur due to skewed X-chromosome inactivation, further highlighting the complexity of X-linked inheritance [120]. To date, no curative therapy is available; however, several approved treatments aim to slow disease progression. In parallel, multiple ongoing clinical trials are designed to target the underlying molecular cause of the disease. These include exon-skipping therapies based on antisense oligonucleotides, which restore the disrupted reading frame in patients with specific dystrophin gene deletions, allowing the production of a truncated yet partially functional dystrophin protein by modulating pre-mRNA splicing. Another promising strategy is adeno-associated virus-mediated systemic gene replacement therapy. However, the large size of the dystrophin gene exceeds the packaging capacity of AAV vectors, necessitating the development of highly abbreviated micro-dystrophin constructs. Several independent clinical trials using these micro-dystrophins are currently underway [121,122].

Beyond dystrophinopathies, limb-girdle muscular dystrophies exemplify the extensive genetic diversity underlying primary muscle disease. LGMDs are caused by pathogenic variants in more than 30 genes inherited in autosomal dominant or recessive patterns and encode proteins essential for membrane stability, muscle regeneration, and intracellular repair mechanisms [123,124]. Frequent subtypes include sarcoglycanopathies, resulting from variants in *SGCA*, *SGCB*, *SGCG*, and *SGCD*, which destabilize the dystrophin–glycoprotein complex, and dysferlinopathies caused by pathogenic variants in *DYSF*, leading to impaired membrane repair and a phenotypic continuum ranging from proximal to distal myopathies [125]. The emergence of gene-based therapies, particularly adeno-associated viral gene transfer approaches, underscores the translational importance of precise molecular diagnosis in this highly heterogeneous group [126]. Myotonic dystrophies introduce a distinct genetic mechanism, in which unstable repeat expansions in noncoding regions result in RNA-mediated toxicity rather than loss of protein function. Myotonic dystrophy type 1, caused by a CTG repeat expansion in the 3′ untranslated region of the *DMPK* gene, demonstrates how expanded RNA transcripts can disrupt splicing regulation and downstream cellular pathways, producing a multisystem disorder with marked phenotypic variability and anticipation across generations [127]. This pathogenic paradigm has catalyzed the development of antisense and small-molecule therapies aimed at neutralizing toxic RNA species and restoring normal RNA processing [128,129]. Congenital myopathies further extend the genetic and biological spectrum of primary muscle disease, linking early-onset weakness and hypotonia to defects in muscle fiber organization, membrane trafficking, and nuclear architecture [130]. Severe forms such as X-linked myotubular myopathy caused by pathogenic variants in *MTM1* highlight the consequences of disrupted phosphoinositide metabolism, while autosomal dominant centronuclear myopathy due to *DNM2* variants and laminopathies associated with *LMNA* mutations illustrate how perturbations in vesicle trafficking and nuclear structure can converge on similar clinical phenotypes [131,132]. These disorders are increasingly becoming targets for nucleotide-based and gene-modulating therapeutic strategies [133].

Metabolic myopathies are a clinically heterogeneous group of disorders characterized by abnormalities in glycogen storage, glycolysis, and lipid metabolism. Together, these conditions illustrate how inherited defects in enzymatic pathways impair muscle energy metabolism and lead to progressive muscle weakness [134]. Pompe disease, caused by biallelic pathogenic variants in the *GAA* gene encoding lysosomal acid α-glucosidase, underscores the importance of molecular diagnosis in guiding prognosis and treatment [135]. Based on age at onset, the disease is classified into infantile-onset Pompe disease (IOPD), which includes the severe classic infantile form, and late-onset Pompe disease (LOPD). In addition, genetic characterization allows for the determination of cross-reactive immunologic material (CRIM) status, a critical determinant of immune response to enzyme replacement therapy [136]. Globally, IOPD occurs in approximately 1 in 150,000 live births and is characterized by early-onset hypotonia, progressive muscle weakness, and hypertrophic cardiomyopathy within the first year of life, with markedly reduced GAA activity in blood and less than 1% residual activity in skin or muscle tissue [137]. In classic infantile-onset Pompe disease, clinical manifestations typically begin within the first months of life, with cardiomyopathy evident before 12 months of age, and the disease progresses rapidly, often leading to respiratory and cardiac failure and, if untreated, death within the first year of life. In contrast, LOPD may present from the first year of life onward and is generally associated with slower disease progression and the absence of hypertrophic cardiomyopathy during the first year of life. Enzyme replacement therapy (ERT) is the current standard of care for Pompe disease and represents a life-saving treatment, particularly in infantile-onset forms. Early initiation of ERT significantly improves cardiac, respiratory, and motor outcomes, leading to increased overall and ventilator-free survival in patients with IOPD. Alglucosidase alfa was the first approved ERT for both IOPD and LOPD, followed by next-generation therapies, including avalglucosidase alfa and cipaglucosidase alfa plus miglustat, which have expanded treatment options, particularly for patients with LOPD, and are under ongoing evaluation for broader clinical use [136,138]. Moreover, several preclinical and early-stage clinical gene therapy studies are currently underway, targeting different tissues and employing a variety of transgenes [139].

Collectively, primary muscle diseases represent not discrete entities but an interconnected genetic landscape in which diverse molecular mechanisms converge on muscle dysfunction. The integration of molecular genetics into pediatric neuromuscular medicine has transformed diagnosis and management, enabling precision medicine approaches that increasingly target the underlying genetic defect or its downstream consequences. As gene-based and nucleotide-based therapies continue to evolve, early and accurate genetic diagnosis remains essential to optimize outcomes and modify the natural history of inherited neuromuscular disorders.

#### 5.2.5. Neurodevelopmental Disorders

Neurodevelopmental disorders (NDDs) represent a broad and highly heterogeneous group of conditions that include intellectual disability (ID), developmental delay (DD), autism spectrum disorder (ASD), and attention-deficit/hyperactivity disorder (ADHD) [140]. These phenotypes show substantial clinical overlap and frequently co-occur, reflecting shared neurodevelopmental pathways. A consistent sex bias is observed across NDDs, with male-to-female ratios ranging from approximately 1.2:1 to 4:1 [141]. Neurodevelopmental presentations are among the most common manifestations of underlying genetic disorders, and current professional guidelines therefore recommend comprehensive genetic evaluation for all individuals with NDDs [29]. From a genomic perspective, NDD etiology is complex and multifactorial, involving contributions from rare and common genetic variants and structural and regulatory genomic alterations. Over the past decade, the widespread application of high-throughput sequencing technologies, particularly WES and WGS, has fundamentally reshaped our understanding of NDD genetic architecture [29]. Large-scale genomic studies have identified more than 200 high-confidence risk genes and susceptibility loci, implicating pathways involved in synaptic function, chromatin remodeling, transcriptional regulation, and neurodevelopment. Despite these advances, currently identified genetic factors account for only approximately 30% of NDD cases, highlighting substantial unresolved genetic complexity [142]. Early genetic investigations relied on cytogenetic approaches, including karyotyping and fluorescence in situ hybridization, which enabled the detection of aneuploidies and large chromosomal rearrangements and led to seminal discoveries such as *FMR1* expansions causing Fragile X syndrome. The introduction of chromosomal microarray analysis substantially increased diagnostic yield by enabling the detection of submicroscopic copy number variations (CNVs), revealing recurrent pathogenic deletions and duplications associated with ASD and ID, including loci at 1q21.1, 7q11.23, 15q11.2, 16p11.2, and 22q11.2 [29]. The advent of NGS enabled the systematic identification of single-nucleotide variants (SNVs), small insertions and deletions, and noncoding variants across the genome [143]. WES and WGS are now central tools for both gene discovery and clinical diagnostics, supported by population reference resources such as the Genome Aggregation Database (gnomAD), which provide critical allele frequency data for variant interpretation [144]. These resources have facilitated the identification of rare and ultra-rare variants under strong negative selection, which are preferentially enriched among individuals with NDDs. Trio-based genomic analyses have further demonstrated that de novo variants represent one of the most frequent and impactful genetic mechanisms underlying NDDs, particularly in sporadic and syndromic presentations. Collectively, these genomic approaches now enable the identification of a molecular diagnosis in more than 40% of individuals with syndromic NDDs. Importantly, genomic diagnoses inform genotype–phenotype correlations and support precision medicine approaches by guiding surveillance strategies and anticipatory management of associated medical comorbidities [145]. Continued expansion of genomic datasets, improved functional annotation of noncoding regions, and integrative multi-omic approaches will be essential to further resolve the remaining genetic heterogeneity of NDDs.

#### 5.2.6. Movement Disorders

Movement disorders comprise a heterogeneous group of neurological conditions characterized by the impaired control and execution of voluntary and involuntary movements. Although traditionally classified according to phenomenology—such as hyperkinetic, hypokinetic, ataxic, or tone-related syndromes, this descriptive approach does not reflect underlying disease mechanisms [146]. With the expansion of genetic diagnostics, etiological classification has become increasingly important, particularly in distinguishing primary (genetic) from secondary (acquired) movement disorders. Two key genetic principles have reshaped the understanding of movement disorders. First, genetic heterogeneity, whereby similar clinical phenotypes can result from pathogenic variants in multiple different genes; and second, phenotypic pleiotropy, in which variants in a single gene can lead to a range of distinct movement disorder phenotypes. These concepts, together with growing insight into disease mechanisms and the limitations of purely symptomatic treatments, have driven a shift toward molecularly targeted, disease-modifying therapies within a precision medicine framework. Friedreich’s ataxia (FRDA) exemplifies this genetic paradigm. FRDA is an autosomal recessive disorder most commonly caused (≈96% of cases) by biallelic expansion of an unstable GAA trinucleotide repeat in intron 1 of the *FXN* gene, resulting in reduced frataxin expression. In the remaining cases, a GAA repeat expansion on one allele is combined with a pathogenic sequence variant or deletion on the other allele. Carriers are asymptomatic, and affected individuals are often isolated cases, although sibling recurrence may occur [147]. FRDA predominantly affects individuals of Indo-Caucasian ancestry, with a molecularly defined prevalence of approximately 0.5 per 100,000 and a carrier frequency estimated between 1 in 60 and 1 in 100. Age at disease onset correlates strongly with the size of the GAA expansion, particularly the shorter allele, with each additional 100 repeats associated with an earlier onset by approximately 2–3 years [148]. While FRDA typically presents in childhood or adolescence, late-onset and very late-onset forms are increasingly recognized. Clinically, FRDA is characterized by progressive ataxia, cerebellar dysfunction, sensory neuropathy, lower limb spasticity, and hypertrophic cardiomyopathy. At the molecular level, impaired frataxin function leads to mitochondrial dysfunction and oxidative stress, which represent central disease mechanisms. The identification of these genetic and molecular pathways has enabled the development of targeted therapies. Omaveloxolone, which modulates cellular responses to oxidative stress through the NRF2 and NF-κB pathways, has recently been approved for the treatment of FRDA in adolescents and adults. As the first, and currently only, approved disease-modifying therapy for FRDA, its approval represents a major milestone in genetically informed treatment for movement disorders. This development underscores the importance of timely recognition of Friedreich’s ataxia and familiarity with its emerging therapeutic options for all pediatric neurologists. Although long-term effects on disease progression remain under investigation, FRDA has emerged as a paradigmatic example of how genetic insight can transform the clinical approach to pediatric movement disorders, reinforcing the central role of molecular diagnosis in guiding prognosis and therapeutic innovation [149].

## 6. Conclusions and Future Directions

Taken together, the rapid advances in genetic and genomic technologies have fundamentally transformed the understanding, diagnosis, and management of pediatric neurological disorders. The widespread implementation of next-generation sequencing has enabled a comprehensive interrogation of genetic architecture underlying many common neurological phenotypes, facilitating earlier and more precise diagnoses and shifting clinical practice from symptom-based classification toward molecularly defined disease entities. Whole-exome and whole-genome sequencing, in particular, have proven invaluable in resolving diagnostically complex or ambiguous cases, providing actionable insights that inform prognosis, guide clinical management, and support genetic counseling for affected families.

Nevertheless, this overview has inherent limitations that warrant careful consideration. Its scope is intentionally centered on the most frequently encountered pediatric neurological conditions in clinical practice and therefore does not encompass the full spectrum of neurogenetic disorders including many rare and ultra-rare conditions. While an increasing number of these disorders can now be identified through exome and genome sequencing approaches, they remain beyond the practical scope of this narrative overview. As a result, the genetic landscape described here likely underestimates the overall diversity and complexity of pediatric neurogenetic disease. In addition, formal, evidence-based guidelines for the optimal indication and use of genetic testing are still lacking for several neurological presentations, leading to variability in clinical practice and reliance on expert opinion and experience from specialized neurogenetics programs. Furthermore, published diagnostic yields should be interpreted with caution, as they are often derived from retrospective cohorts and may be influenced by referral bias, study design, and the underrepresentation of diverse populations.

Importantly, the clinical utility of genetic and genomic testing extends beyond diagnosis alone. Molecular diagnoses increasingly influence therapeutic decision-making, enable access to gene-specific and mechanism-based treatments, and support enrollment in clinical trials of emerging gene- and nucleotide-based therapies. In this context, precision medicine is no longer solely a future aspiration: in selected pediatric neurological disorders, gene-based and molecular therapies have already entered clinical practice and are actively reshaping disease trajectories. A paradigmatic example is SMA, in which gene replacement and splicing-modulating therapies have profoundly changed the natural history of the disease, transforming a previously fatal condition into a treatable disorder when diagnosed early. At the same time, the growing scale and complexity of genomic data introduce challenges related to variant interpretation, data storage, and integration into routine clinical workflows. Beyond these scientific and clinical challenges, ethical and health-system considerations are increasingly critical in pediatric genomic medicine. The management of incidental findings represents a central concern, as sequencing may reveal pathogenic variants un-related to the primary indication. Interpretation of genetic data in children is complex, given evolving phenotypes and uncertainty regarding disease onset, requiring informed consent that addresses unique pediatric vulnerabilities. Equitable access remains a major barrier, with significant racial and socioeconomic disparities in genetic testing completion, though innovative care models, including telegenetics, offer potential solutions. Addressing these ethical and systemic challenges is essential to ensure responsible implementation and maximize patient benefit [150,151]. In parallel, emerging therapeutic strategies are expanding the future landscape of pediatric genomic medicine. Gene-editing approaches, including CRISPR-based strategies, represent an important future direction, as technologies such as CRISPR-Cas9 and its variants, including base editing and prime editing, enable precise genetic modifications with high specificity and efficiency, offering the potential to correct disease-causing mutations at their source. Clinical applications are advancing rapidly, supported by the recent regulatory approval of the first CRISPR-based therapy for sickle cell disease and encouraging pre-clinical results in pediatric neurological disorders. However, important challenges remain, including the risk of off-target effects, optimization of delivery systems, and the possibility of pathological immune responses [152,153].

Furthermore, long-term outcomes, durability, and safety of gene-based therapies require careful and continued evaluation. While transgene expression following viral vector delivery may persist for more than a decade, immune responses to vectors can limit therapeutic efficacy and may prevent re-dosing. Additional safety concerns include immune-mediated liver injury, particularly in children receiving high-dose recombinant adeno-associated viral vectors, and potential genotoxicity related to vector integration. Continued research and systematic long-term follow-up will therefore be essential to optimize these therapies and ensure their safe implementation in pediatric populations. Addressing these challenges, scientific, clinical, and ethical, will require sustained interdisciplinary collaboration among neurologists, clinical geneticists, bioinformaticians, and laboratory scientists, as well as continued investment in education and infrastructure.

As sequencing technologies continue to evolve and become more accessible, the diagnostic yield and clinical impact of genomic testing in pediatric neurology are expected to expand further. Future efforts should focus on integrating comprehensive genomic data with deep phenotyping and longitudinal follow-up, developing standardized testing strategies, and ensuring equitable access to advanced genomic technologies. An important challenge in pediatric neurological disorder research is missing heritability, the portion of genetic risk not explained by currently known variants. Future studies may address this gap through WGS to identify rare and noncoding variants, the investigation of genetic modifiers and epigenetic mechanisms, multi-omics integration, and the inclusion of diverse or isolated populations, helping to uncover novel disease genes and clarify the genetic architecture underlying pediatric neurological disorders [154]. By enabling early diagnosis and timely access to gene and mechanism targeted treatments, genomic medicine holds the potential not only to improve outcomes but, in selected conditions, to fundamentally modify or even prevent disease progression. Through these coordinated efforts, the promise of precision medicine, delivering individualized, gene-informed care, can be more fully realized for children with neurological disorders and their families.

## Data Availability

Data will be available on request to the corresponding author.
